# The *de novo* genome assembly and annotation of a female domestic dromedary of North African origin

**DOI:** 10.1111/1755-0998.12443

**Published:** 2015-07-24

**Authors:** Robert R. Fitak, Elmira Mohandesan, Jukka Corander, Pamela A. Burger

**Affiliations:** ^1^ Institut für PopulationsgenetikVetmeduni ViennaVeterinärplatz 1Vienna1210Austria; ^2^ Department of Mathematics and StatisticsUniversity of HelsinkiHelsinkiFIN‐0014Finland; ^3^ Present address: Department of BiologyDuke UniversityDurhamNC27708USA

**Keywords:** adaptation, *Camelus dromedarius*, demography, domestication, next‐generation sequencing

## Abstract

The single‐humped dromedary (*Camelus dromedarius*) is the most numerous and widespread of domestic camel species and is a significant source of meat, milk, wool, transportation and sport for millions of people. Dromedaries are particularly well adapted to hot, desert conditions and harbour a variety of biological and physiological characteristics with evolutionary, economic and medical importance. To understand the genetic basis of these traits, an extensive resource of genomic variation is required. In this study, we assembled at 65× coverage, a 2.06 Gb draft genome of a female dromedary whose ancestry can be traced to an isolated population from the Canary Islands. We annotated 21 167 protein‐coding genes and estimated ~33.7% of the genome to be repetitive. A comparison with the recently published draft genome of an Arabian dromedary resulted in 1.91 Gb of aligned sequence with a divergence of 0.095%. An evaluation of our genome with the reference revealed that our assembly contains more error‐free bases (91.2%) and fewer scaffolding errors. We identified ~1.4 million single‐nucleotide polymorphisms with a mean density of 0.71 × 10^−3^ per base. An analysis of demographic history indicated that changes in effective population size corresponded with recent glacial epochs. Our *de novo* assembly provides a useful resource of genomic variation for future studies of the camel's adaptations to arid environments and economically important traits. Furthermore, these results suggest that draft genome assemblies constructed with only two differently sized sequencing libraries can be comparable to those sequenced using additional library sizes, highlighting that additional resources might be better placed in technologies alternative to short‐read sequencing to physically anchor scaffolds to genome maps.

## Introduction

The dromedary (*Camelus dromedarius*) is the most common of all *Camelus* species and is easily distinguished from its congeners, the Bactrian (*Camelus bactrianus*) and wild (*Camelus ferus*) camels, by the presence of a single hump. Dromedaries are widespread throughout northern and eastern Africa, the Arabian Peninsula and southwest Asia, and a large feral population exists in Australia (Köhler‐Rollefson 1991; Spencer & Woolnough 2010). Throughout their range, dromedaries are bred for a multitude of purposes including meat, milk production, transportation, wool and sport (Bulliet 1990; Grigson 2012). Archaeozoological evidence suggests that the domestication of dromedaries took place between 3000 and 4000 years ago in the east coast of the Arabian Peninsula (Uerpmann & Uerpmann 2002). Unlike many other domestic livestock, the wild ancestor of dromedaries is extinct, and despite the examination of ancient wild dromedary remains, a formal taxonomic description of the extinct species has not been made.

In addition to the economic importance, dromedaries harbour an assortment of biological and physiological traits specifically adapted to extreme heat and harsh, desert conditions. For example, dromedaries do not begin to sweat until body temperatures reach as high as 42 °C, can tolerate fluctuating body temperatures as much as 6 °C and withstand water loss >30% of their body mass (see Köhler‐Rollefson 1991 for a review). Furthermore, studies have uncovered several camel products with applications in human medicine, including unique immunoglobulin molecules that are useful in nanobody technology (Muyldermans *et al*. 2009) and milk that may contain beneficial properties for the treatment of diabetes (Agrawal *et al*. 2011).

As a result of increased economic, medical and evolutionary value of camels, understanding the genetic basis of these and other relevant traits is necessary. However, unlike many other livestock species (e.g. cow, horse, pig), genetic and genomic resources for camels, especially dromedaries, are lacking. Recent work has provided the first complete genome sequence of the dromedary (Wu *et al*. 2014), and additional genomes from its congeners have also been made available (Jirimutu *et al*. 2012; Burger & Palmieri 2014; Wu *et al*. 2014). These studies have identified candidate loci responsible for various adaptations to desert conditions, insulin resistance and camels’ unique immune system. Although interspecific comparative genomics in camels have proven useful, little knowledge regarding the intraspecific variation, especially in dromedaries, exists. Large‐scale analyses of genetic variation, or polymorphisms, within a species or population can uncover additional candidates for selection through dense genome scans of population divergence or hitchhiking (Ellegren 2008). For example, genome‐wide analysis of single‐nucleotide polymorphisms (SNPs) in cattle has identified loci linked to milk production traits (Pryce *et al*. 2010) and this knowledge has been implemented in breeding programmes designed to improve production traits through the process of genomic selection (reviewed by Hayes *et al*. 2009; Schefers & Weigel 2012). Furthermore, these genomic scans of polymorphism can inform assessments of demographic history, where population bottlenecks and small population sizes, often associated with mammalian megafauna, can obscure the ability to detect patterns of selection in genomes (Akey *et al*. 2004; Pool *et al*. 2010).

In this study, we sequenced and assembled a second genome for the dromedary. The individual's (‘Waris’) origin can be traced back to North Africa and the Canary Islands. Both regions are genetically distinct from populations in southern Arabia and further east, yet are indistinguishable from one another despite dromedaries from the Canary Islands having been isolated since the fifteenth century (Schulz *et al*. 2010). We quantitatively compared our dromedary genome and its demographic history with the existing reference and identified SNPs useful for future studies on the evolutionary and agricultural importance of this species. Finally, we comment on the data availability and transparency of bioinformatic methods for next‐generation sequencing studies and present our methods and results consistent with current recommendations (Whitlock 2011).

## Materials and methods

### Sample collection, sequencing and assembly

Whole blood from a female dromedary named ‘Waris’ living at the First Austrian Camel Riding School in Eitental, Austria, was collected during a routine veterinary examination, and an aliquot was used for genomic DNA extraction with the MasterPure DNA Purification Kit (Epicenter, USA). The mother of Waris originated from the population on the Canary Islands, whereas the father was of North African origin. A 500‐bp insert paired‐end library and a 5‐kb mate‐pair library were prepared and sequenced using three lanes and one lane, respectively, on an Illumina HiSeq 2000 system (Illumina, USA). Preprocessing of the sequence reads included the removal of adapter sequences and removal of reads with >10% uncalled bases and/or >50% of bases with a Phred‐scaled quality score <4. After preprocessing, all 100‐bp (paired‐end) and 50‐bp (mate‐pair) reads were retained as the set of ‘raw’ reads. We trimmed the 3′ end of all raw reads using a modified Mott algorithm in popoolation v1.2.2 (Kofler *et al*. 2011) to a minimum quality score of 20 and a minimum length threshold of 50 bp and 30 bp for the paired‐end and mate‐pair reads, respectively.

We corrected the trimmed, paired‐end reads for substitution sequencing errors using quake v0.3.5 (Kelley *et al*. 2010). Salzberg *et al*. (2012) showed previously that the error correction of sequencing reads can greatly improve the *de novo* assembly of genomes, including genomes assembled using the program abyss (Simpson *et al*. 2009). quake uses the distributions of infrequent and abundant *k*‐mers to model the nucleotide error rates and subsequently corrects substitution errors. As input to quake and again after error correction, we counted the frequency of 20‐mers in the paired‐end reads using dsk v1.6066 (Rizk *et al*. 2013). To estimate genome size, we divided the total number of error‐free 20‐mers by their peak coverage depth.

We assembled the genome using the trimmed and error‐corrected paired‐end reads with abyss v1.3.6. To determine the optimal *k*‐mer length, we repeated the assembly using *k *=* *40–88 in 8‐bp increments. All scaffolding steps were performed using the trimmed mate‐pair reads also in abyss, and only scaffolds longer than 500 bp were retained. We evaluated the completeness of each assembly using cegma v2.4 (Parra *et al*. 2007) with the ‘–mam’ parameter for mammalian intron structure. cegma annotates highly conserved, core eukaryotic genes (CEGs) that should be present in the genome.

From the resulting assemblies, we selected two (the one with the fewest scaffolds, *k *=* *48, and the one with the longest N50, *k *=* *64) for further evaluation in reapr v1.0 (Hunt *et al*. 2013). reapr evaluates the accuracy of an assembly through the identification of small, local errors (single base substitutions and short insertions/deletions) and mis‐assemblies (such as structural or scaffolding errors) using mapped, paired‐end reads. One of the primary metrics calculated by reapr is the fragment coverage distribution (FCD). This statistic is measured on a per‐site basis and is the distribution of coverage depth for fragments (regions between the outermost ends of a set of properly paired reads) containing the base. The difference between the observed FCD and its theoretical distribution is the FCD error, and strings of bases with high FCD error indicate assembly mistakes (Hunt *et al*. 2013). The FCD error cut‐off for calling a failed region was determined automatically in reapr after randomly sampling 10^5^ windows of 100 bp in length. The scaffolds are cleaved at these locations to produce a ‘broken assembly’ more useful for comparison. As recommended input into reapr, we mapped the trimmed and error‐corrected paired‐end reads to each genome assembly using smalt v0.7.0.1 (https://www.sanger.ac.uk/resources/software/smalt) with default parameters. To assess the effects of error‐correcting reads prior to assembly, we repeated the assembly (*k *=* *48 and *k *=* *64), cegma and reapr analyses as described above using the trimmed (uncorrected) paired‐end reads.

We selected the assembly with the highest proportion of error‐free bases, fewest FCD errors and the longest N50 in the broken assembly. We assessed the composition of the short (<500 bp) scaffolds omitted from the final assembly using a blastn v2.2.30 (http://ncbi.nlm.nih.gov/blast) search against the nucleotide database of GenBank with an e‐value cut‐off of 10^−3^. The genome assembly is available in GenBank as Accession no. GCA_000803125.1.

### Comparison with existing dromedary genome

We further assessed the quality of our genome assembly through comparison with the recently published dromedary reference (Wu *et al*. 2014) (GenBank Accession no. GCA_000767585.1). We downloaded the raw reads for the three short‐insert libraries (170‐, 500‐ and 800‐bp inserts) from the reference assembly. As described in Wu *et al*. (2014), we removed reads with >5% uncalled bases, with >40 bases of Phred‐scaled quality ≤20, with adapter contamination (match length ≥10 bp, mismatch ≤3 bp), with duplicated forward and reverse pairs and with overlapping forward and reverse pairs (excluding the 170‐bp insert library, overlap ≥10 bp, mismatch ≤10% bp). We then error‐corrected 17‐mers that only occurred once (Wu *et al*. 2014) and repeated the reapr pipeline separately for each library as described above.

In addition, we performed a separate whole‐genome alignment of both complete dromedary genome assemblies using mugsy v1.2.3 (Angiuoli & Salzberg 2011) with a maximum distance of 500 bp for chaining anchors into locally collinear blocks. The final alignment blocks were filtered using maffilter v1.1.0 (Dutheil *et al*. 2014) with the following criteria: using a sliding window of 10 bp, we excluded the window from the alignment if more than five gaps (including ‘N’) were present and subsequently split the block. We retained alignment blocks with a minimum length of 500 bp.

### Genome annotation

We employed a two‐pass, iterative procedure using the maker v2.31.6 pipeline (Cantarel *et al*. 2008; Holt & Yandell 2011) to manage and evaluate the different evidences for gene annotation. For the first pass, we predicted genes using SNAP (Korf 2004) with hidden‐Markov models developed from the CEGs identified from cegma and an *ab initio* prediction of genes from genemark‐es (Lomsadze *et al*. 2005). This first pass also included alignments from existing dromedary ESTs (Al‐Swailem *et al*. 2010) and protein‐based homology from a concatenated set of Bactrian camel (Accession no. GCF_000311805.1), alpaca (Accession no. GCF_000164845.1) and cow (Accession no. GCF_000003055.4) protein sequences. For the second pass, we predicted genes using both SNAP and augustus v2.5.5 (Stanke *et al*. 2006), both trained with a hidden‐Markov model developed from the predictions of the first maker pass. The second pass also included the EST‐ and protein‐based evidence as described in the first pass. All runs of maker included the masking of repetitive regions using repeatmasker v4.0.3 (Smit *et al*. 1996) against the repbase v19.07 (Jurka *et al*. 2005) library. For each gene prediction, we selected the evidence with an annotation edit distance (AED) < 0.75.

Using the longest isoform for each protein sequence, we functionally annotated each gene using a combination of blastp v2.2.30 (http://ncbi.nlm.nih.gov/blast) and interproscan 5.7.48 (Jones *et al*. 2014). blast searches were performed against metazoan protein sequences from the ‘nr’ database with an e‐value cut‐off of 10^−3^, and only the top 20 hits were retained. We used interproscan to assign protein domains and motif to sequences through comparison against a variety of databases (i.e. tigrfam, prodom, smart, hamap, prositepatterns, superfamily, prints, panther, gene3d, pirsf, pfama, prositeprofiles, coils). Annotations were stored as Gene Ontology (GO) terms for each sequence. Next, we used the protein sequences to identify single‐copy orthologs shared with the *C. ferus* (GCA_000311805.2) and with the *Bos taurus* (Accession no. GCF_000003055.5) genomes using orthomcl (Li *et al*. 2003). We used a minimum identity of 30% and an e‐value cut‐off of 10^−5^ to call orthologs.

In combination with the homology‐based repeat annotation described above, we also characterized *de novo* repetitive elements from the sequencing reads and genome assembly using separate approaches. To identify repeats directly from the trimmed and error‐corrected paired‐end reads, we used the method implemented in repark v1.2.1 (Koch *et al*. 2014). This method works by generating a *de novo* assembly of the abundant *k*‐mers (*k *=* *31) in the reads. repark determined the threshold for defining abundant 31‐mers by fitting a linear function to the slope of the descending segment of the Poisson‐like unique *k*‐mer fraction (Fig. S1, Supporting information). The abundant 31‐mers were defined as those occurring at frequency greater than twice the *x*‐intercept of the linear function. The *x*‐intercept of our linear function was 49, and therefore, abundant 31‐mers were defined as those occurring more than 98 times in the sequencing reads. The abundant 31‐mers were assembled with velvet v2.0 (Zerbino & Birney 2008). We calculated statistics for the contigs using quast v2.3 (Gurevich *et al*. 2013). We identified and classified repeat families for both assemblies (the repetitive 31‐mers and the genome assembly) using a combination of recon v1.08 (Bao & Eddy 2002) and repeatscout v1.0.5 (Wootton & Federhen 1993; Benson 1999). Final repeat libraries for each assembly were subsequently built using repeatmodeler v2.1 (Smit & Hubley 2008).

The noncoding RNA genes were predicted with structure‐based homology search by infernal v1.1.1 (Nawrocki *et al*. 2009) against the rfam database (Release 12.0) (Griffiths‐Jones *et al*. 2003). We used a ‘gathering’ cut‐off score of 85% for the covariance models and a confidence threshold (e‐value) of 10^−9^. We annotated CpG islands using the ‘cpgplot’ tool in emboss v6.5.7 (Rice *et al*. 2000) with the repeat‐masked genome employing a window length of 100 bp, a minimum island length of 200 bp, minimum GC content of 0.5 and a minimum average observed ratio of C+G to CpG of 0.6.

### Variant identification and demographic analysis

We aligned the trimmed and error‐corrected paired‐end reads back to the final genome assembly using bwa v0.6.2 (Li & Durbin 2009). From the alignment, we removed duplicated reads and filtered all alignments to contain only unambiguously mapped and properly paired reads using samtools v1.1 (Li *et al*. 2009). We identified variants (SNPs and insertion/deletion polymorphisms) using a combination of samtools and platypus (Rimmer *et al*. 2014). Both of these variant callers have been shown to produce reliable results for single‐sample SNP calling and do not require preprocessing steps that realign reads around indels and recalibrate base quality scores (Liu *et al*. 2012; Baes *et al*. 2014). As recommended by Baes *et al*. (2014), we included the consensus set of variants identified by both methods. We further excluded variants with a Phred‐scaled quality score <20, that were within five base pairs of another variant, and whose depth of coverage was less than 1/3 or more than twice the mean genome coverage of the alignment. The quality of the final set of variants was assessed using the ratio of transitions (pyrimidine ↔ pyrimidine or purine ↔ purine) to transversions (purine ↔ pyrimidine) in vcftools v0.1.12b (Danecek *et al*. 2011). This ratio, called the ti/tv ratio, is known to be ~2.1 in human genomes and is often used to evaluate variant prediction quality (DePristo *et al*. 2011; Liu *et al*. 2012; Baes *et al*. 2014). The SNP density within the genome and divergent sites from alignment with the reference genome were estimated using nonoverlapping 1000‐bp windows and then separately for the annotated regions (*i.e*. exons, introns, CpG islands, repetitive regions) in vcftools.

We examined the historical changes in effective population size (N_e_) of the dromedary genome using the pairwise sequentially Markovian coalescent model (psmc v0.6.4) (Li & Durbin 2011). psmc infers N_e_ at a given time in the past from a single diploid individual using the rates of coalescence events across the genome. Because psmc is highly dependent on the density of polymorphic sites, we performed two different runs of psmc: (i) using only the sites with a mean mapping quality ≥20 and coverage between one‐third and twice the mean genome coverage (lenient conditions) and (ii) a consensus genome sequence generated from our filtered set of variants described above and with repetitive regions masked (strict conditions). Both analyses in psmc were performed for 25 iterations using ‐p and ‐t parameters chosen manually to infer ~10 recombination events in the interval (Li & Durbin 2011) and an initial theta/rho ratio (−*r*) of 5. The variance was assessed using 100 bootstrap replicates, and final estimates of N_e_ and time were scaled with a mutation rate of 2.5 × 10^−8^ and a generation time of five years.

## Results and discussion

### Sequencing and assembly comparisons

We sequenced the genome of a female dromedary of North African ancestry, ‘Waris’, using only one short‐insert (500 bp) and one long‐insert (5 kb) library. Prior to error‐correcting reads, these shotgun libraries generated 66.4× coverage of the genome. A summary of the sequencing reads and estimated genome coverage can be found in Table 1. We counted the frequency of unique 20‐mers in the trimmed paired‐end reads and, using 20‐mers with a frequency of three or less, determined the rate of base substitution error to be 2.7% (Table S1, Supporting information). This error rate is higher than that commonly reported for the Illumina HiSeq 2000 system (0.1%–1%) (Glenn 2011; Minoche *et al*. 2011) and may be the result of reduced sequencing performance and/or the presence of low‐abundance, contaminating sequence (e.g. humans). For instance, the extracted DNA was from whole blood, which may contain a wide variety of microorganisms whose DNA abundance is rare relative to the host. As suggested by Salzberg *et al*. (2012), we corrected reads for these errors (Fig. S2, Supporting information) and found only a 1.6% reduction in the total number of bases used for assembly (~1× coverage reduction in the final assembly) (Table 1). A majority of corrections were made to bases with a Phred‐scaled quality score <10 and were consistent between forward, reverse and unpaired reads (Fig. S3, Supporting information). Using the counts of 20‐mers with a frequency >3 and a peak coverage of 35x, we estimated the genome size to be 2.25 gigabases (Gb). This estimate is similar to that reported previously for the dromedary (2.27 Gb) using the frequency of 17‐mers (Wu *et al*. 2014) but less than that reported using flow cytometry (2.56 Gb; Krishan *et al*. 2005).

**Table 1:** Read statistics after quality and length trimming

Library	# Reads with partner	# Reads without partner	Mean length (SD)	Total number of bases	Sequence coverage
500‐bp PE	579 823 726	5 045 754	98.2 (6.4)	114 374 878 323	55.7×
500‐bp PE‐corrected	562 416 289	22 102 005	98.1 (7.0)	112 536 342 122	54.8×
5‐kb MP	224 408 840	2 834 348	48.6 (1.8)	21 970 012 359	10.7×
Total (Corrected+MP)	786 825 129	24 936 353	—	134 506 354 481	65.5×

PE, paired‐end library; MP, mate‐pair library.

**Figure 1:**
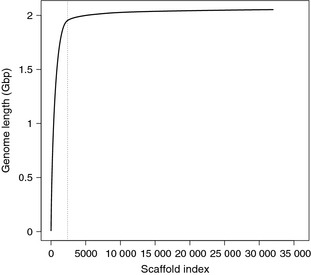
Cumulative length of the African *Camelus dromedarius* assembly. Scaffolds are sorted from longest to smallest along the horizontal axis. The vertical dotted line indicates the number of scaffolds containing 95% of the total assembly.

We compared different *k*‐mer sizes for assembly of the trimmed and error‐corrected paired‐end reads (Fig. S4, Supporting information) and found that *k *=* *48 produced the fewest scaffolds (24 058) and most CEGs (99.1%), whereas *k *=* *64 produced the longest N50 (1 482 444 bp) and longest scaffold (9 719 801 bp). A quantitative comparison of these two assemblies both before and after error correction revealed that the use of error‐corrected reads produced assemblies with more error‐free bases and fewer gaps, FCD errors and collapsed repeats (Table S2, Supporting information). Error‐correcting reads also generated broken assemblies with longer N50 values (Table S2, Supporting information). We selected the assembly using corrected reads and *k *=* *64, which outperformed the other assemblies in a variety of metrics given in Table S2 (Supporting information) (e.g. most error‐free bases, fewest FCD errors, fewest gaps, longest N50 in the broken assembly). The final assembly was 2.06 Gb and contained 35 752 scaffolds (≥500 bp) with a GC content of 41.3% (Table 2). We omitted ~4.1 million small scaffolds (<500 bp) from the assembly, a majority (66.6%) of which either had no databases matches or were excluded from searches by the default low‐complexity filter in BLAST. Of the remaining small scaffolds with a database match, ~1.1 million (80.2%) were *C. dromedarius* microsatellite sequences and the rest were distributed among other species, especially *Vicugna pacos*,* Sus scrofa* and *Homo sapiens* (Fig. S5, Supporting information). The N50 of the assembly was 1.48 megabases (Mb), and 95% of the assembly was contained in the longest 2379 scaffolds (Fig. 1). We annotated 452 (98.7%) CEGs, indicative of the completeness of the assembly.

**Table 2:** Summary of the dromedary genome assembly presented in this study compared with the current reference

	*k *=* *64‐C African dromedary	Reference^a^ Arabian dromedary
# Scaffolds	35 752	32 572
Mean length (bp)	57 481.1	61 526.7
Total length (bp)	2 055 063 633	2 004 047 047
Longest (bp)	9 719 801	23 736 781
GC content	41.3%	41.2%
Repeat content	33.7%	28.4%
N50 (count)	1 482 444 (393)	4 188 677 (132)
N60 (count)	1 108 832 (553)	2 993 967 (190)
N70 (count)	842 144 (764)	2 137 136 (268)
N80 (count)	558 658 (1063)	1 311 427 (389)
N90 (count)	260 185 (1592)	689 795 (594)
Number of gaps	150 386	72 775
Total gap length	53 439 631	22 596 073
CEGs^b^	98.7%	98.5%

a Accession no. GCA_000767585.1; Wu *et al*. (2014).

b Proportion of 458 core eukaryotic genes (CEGs) identified using cegma.

**Figure 2:**
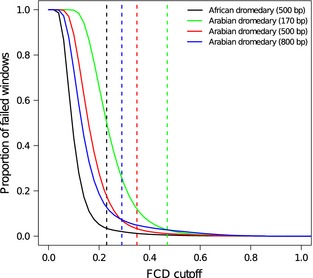
Calculation of the fragment coverage distribution (FCD) error cut‐off. For each potential FCD cut‐off, each solid line represents the proportion of 100‐bp windows that would fail and subsequently be labelled as an assembly error. The vertical dashed lines are the cut‐off scores determined in reapr using the value where the normalized (between −1 and 1) first and second derivatives are ≥0.05. See Hunt *et al*. (2013) for a complete description of the method. The colours correspond with the different read alignments separated by genome and insert size.

Many assembly characteristics (e.g. number of scaffolds, mean scaffold length, GC content, repeat content, CEGs identified) were markedly similar to the current dromedary reference genome, suggesting that the *C. dromedarius* genome sequences are relatively robust to the assembly method used. Our assembly did have a shorter scaffold distribution than the current reference (N50 = 1.48 Mb compared with 4.2 Mb, respectively) and contained twice as many gaps (150 386 compared with 72 775, respectively) (Table 2). Because N50 and other scaffold length metrics are not necessarily indicative of assembly quality (Bradnam *et al*. 2013; Hunt *et al*. 2013), we quantitatively compared our assembly with the existing reference using trimmed and error‐corrected paired‐end reads mapped to the genome sequence (see Table S3, Supporting information for read Accession nos). Our genome consistently had a larger proportion of error‐free bases (91.8%), fewer FCD errors (37 015) and fewer reads in the wrong orientation (113 677), whereas the reference assembly often contained fewer collapsed repeats (Table 3). Furthermore, the cut‐off for defining FCD errors in our assembly was more stringent than in the reference, and when comparing the same cut‐off, fewer windows were called as errors (Fig. 2). These results support that traditional assembly statistics (e.g. N50, mean length, number of scaffolds) do not necessarily indicate the quality and suggest that more robust quantitative comparisons should be performed. For example, the method employed by Wu *et al*. (2014) to assemble the reference genome has been shown to artificially inflate scaffold lengths at the expense of increasing assembly errors (Salzberg *et al*. 2012; Bradnam *et al*. 2013).

**Table 3:** Frequency of different assembly errors compared with the reference genome for short‐insert reads (separated by insert size)

	*k *=* *64‐C African dromedary	Reference^a^ Arabian dromedary		
Insert size	500 bp	170 bp	500 bp	800 bp
Error‐free bases	91.8%	83.4%	74.9%	68.6%
FCD^b^ errors	37 015	9 641 002	203 806	195 429
Collapsed repeats	10 233	86 488	8694	4659
Wrong read orientation	113 677	95 230	215 821	210 951

a Accession no. GCA_000767585.1; Wu *et al*. (2014).

b Fragment coverage distribution.

An alignment of the two genomes produced 291 611 blocks with a total alignment length of 1.91 Gb. The mean block length was 6549.4 bp (SD 7006.0 bp) (Fig. S6, Supporting information). This result further supports the high degree of similarity between the genome sequences despite different assembly strategies.

### Genome annotation

We utilized a combination of *ab initio* and evidence‐based homology to identify and annotate protein‐coding elements in the genome. Not accounting for multiple isoforms, we predicted a total of 21 167 genes containing either protein‐ or EST‐based evidence (Fig. S7, Supporting information); a number similar to that reported for the dromedary reference genome (20 714; Wu *et al*. 2014). Nearly, all genes (98.7%) returned a significant match to known metazoan protein sequences, often with high similarity (Fig. S8A, Supporting information). A majority of the top hits for each gene matched other camelid sequences such as *C. ferus* (57.9%) and *Vicugna pacos* (14.1%) (Fig. S8B, Supporting information). We added functional annotations to 17 779 (84.0%) sequences using INTERPROSCAN. A total of 32 965 GO terms were also mapped to 13 198 sequences (Fig. S9, Supporting information). Both the number of single‐copy orthologs and their mean amino acid identity were higher when compared with the *C. ferus* genome (12 170 and 95.1%, respectively) than when compared with the *B. taurus* genome (11 625 and 86.3%, respectively) (Fig. S10, Supporting information). A comparison with the dromedary reference genome was not possible because, at the time of writing, annotation data remained unavailable. Because the annotation pipeline we used was designed to promote future reannotation as more data become available, the accuracy in gene predictions can easily be increased over time.

We estimated 33.7% of our genome to be composed of repetitive elements using a combination of homology‐based and *de novo* approaches (Tables S4 and S5 and Fig. S11, Supporting information). The homology‐based search identified 31.7% of the genome as repetitive, whereas the *de novo* methods based upon the sequencing reads or the assembly predicted less (13.23% and 24.39%, respectively). Only ~2% of the combined set of repetitive elements were specific to the *de novo* approaches which included primarily LINE1 retrotransposons and unclassified repeats (Table S5, Supporting information). Overall, LINE elements accounted for 19.3% of the genome (Fig. S11, Supporting information). We found a total of 3691 noncoding RNA loci (Table S6, Supporting information), including 1369 micro RNAs, 966 small nuclear RNAs and 524 small nucleolar RNAs. We classified 57 708 putative CpG islands that had a mean length of 326.3 (SD 154.1) bases.

### Variant identification and demographic analysis

We mapped 94.1% of the trimmed and error‐corrected paired‐end reads to our genome assembly. After quality control and filtering, 75.8% of the reads were retained resulting in a mean alignment coverage of 40.8x. We identified a set of ~1.4 millions SNPs and 162 538 insertion/deletion polymorphisms that overlapped between the two SNP‐calling algorithms and passed our filtering criteria. The ti/tv ratio for our final set of SNPs was 2.31, consistent with the ratio reported in dairy cattle using the same algorithms and characteristic of a low rate of false‐positive SNPs (DePristo *et al*. 2011; Baes *et al*. 2014).

**Figure 3:**
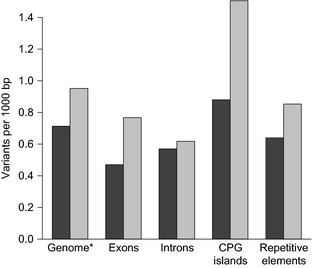
Density of SNPs within the African dromedary genome assembly (dark grey bars) and density of divergent sites (light grey bars) from the alignment with the reference genome (Accession no. GCA_000767585.1). * Genome‐wide density is based upon 1000‐bp nonoverlapping windows.

Across the genome, mean SNP density (heterozygosity) was 0.71 × 10^−3^ (SD 1.4 × 10^−3^), slightly less than reported for the Arabian dromedary (0.74 × 10^−3^; Wu *et al*. 2014). This reduction may be the result of either technical differences in SNP calling (e.g. the method or filtering criteria used) or the consequence of demographic events (e.g. smaller effective population size, increased inbreeding) in the North African/Canary Island population. We suspect the former, considering that for microsatellite data, the Arabian dromedary has a higher *F*_IS_ and lower levels of both observed heterozygosity and allelic richness than dromedaries from North Africa and the Canary Islands (Schulz *et al*. 2010). Nonetheless, SNP density in dromedaries appears to be substantially less than that reported for domestic Bactrian and wild camels (1.0–1.29 × 10^−3^; Jirimutu *et al*. 2012; Burger & Palmieri 2014; Wu *et al*. 2014). Within the dromedary genome, SNP density was highest in CpG islands (0.88 × 10^−3^). This is consistent with the hypermutability of CpG residues (Coulondre *et al*. 1978; Ehrlich & Wang 1981; Sved & Bird 1990) and the positive relationship between mutation rate and CpG content (Walser & Furano 2010). SNP density was lowest in exons (0.47 × 10^−3^) and at intermediate levels in both introns (0.57 × 10^−3^) and repetitive elements (0.64 × 10^−3^) (Fig. 3). Because we omitted SNPs with excessively high coverage, SNP density in repetitive regions may be underestimated.

An alignment with the reference genome produced more than 1.7 million divergent sites, of which 631 468 (36.1%) were biallelic SNPs and the remaining sites were either insertion–deletion polymorphisms or uncalled bases. Nearly, all of these biallelic SNPs (99.4%) overlapped with the SNPs identified within our genome assembly. The relative density of divergent sites across different elements of the genome was similar to the density of SNPs (Fig. 3), with the exception of introns, which contained fewer divergent sites (0.62 × 10^−3^) than exons (0.77 × 10^−3^). Because introns are expected to contain more variation than exons, this result may be the product of increased alignment ambiguity and subsequent filtering of the more variable regions. The density of divergent sites was also markedly higher within CpG islands (1.51 × 10^−3^) than in all other genomic regions (Fig. 3).

**Figure 4:**
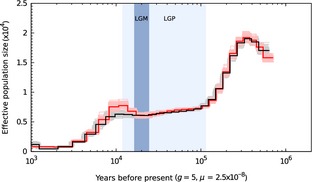
Historical effective population size of the African dromedary inferred with the filtered, repeat‐masked set of variants (black line; strict conditions) and with default parameters (red line; lenient conditions) in psmc (Li & Durbin 2011). The lighter‐coloured lines of the same colour represent the 100 bootstrap replicates. The result is scaled using a generation time (g) of five years and a per‐base mutation rate (*μ*) of 2.5 × 10^−8^. The light‐blue and blue‐shaded regions indicate the last glacial period (LGP) and last glacial maximum (LGM), respectively.

We examined the historical demography using the psmc model and found consistent histories with little variance among both lenient and strict conditions (Fig. 4). Both conditions had a maximum N_e_ of ~20 000 approximately 350 thousand years before present (kybp) with a substantial bottleneck suffered thereafter. This bottleneck reduced N_e_ by nearly 70% during the ~ 300–100 kybp interval leading up to the last glacial period (LGP). The N_e_ declined gradually during the LGP between 100 and 20 kybp. At this time, at the end of the last glacial maxima (LGM; ~20 kybp), the lenient and strict conditions indicated either a small increase or constant N_e_, respectively, until a second, more recent, bottleneck further reduced N_e_ to <1000 individuals beginning 10 kybp. The number of coalescent events occurring more recently than ~1 kybp is inadequate to accurately infer demographic history from this period. This pattern of climate‐driven demographic changes has been observed in a variety of mammalian megafauna (Lorenzen *et al*. 2011; Orlando *et al*. 2013; Wu *et al*. 2014), although anthropogenic effects may have played a critical role in the most recent population reduction. More extensive surveys of camel remains in the archaeological record would be required to disentangle the roles of climate change and humans in driving the decline in dromedary population size. Unfortunately, Wu *et al*. (2014) did not report the generation time or mutation rate used to scale the demographic history of the Arabian dromedary, thus preventing a more thorough comparison with our result.

## Conclusions

Here, we reported a second dromedary genome sequence that provides additional genetic resources from a geographically distinct region. Our results demonstrated that draft genome assemblies constructed using only one short‐ and one long‐insert sequencing libraries can be comparable to those sequenced using more than two library sizes (e.g. 6 in this comparison). This suggests that rather than sequencing numerous libraries of various sizes resources are better spent on physically mapping the genome or on different technologies. For example, methods such as optical mapping (Chamala *et al*. 2013; Shearer *et al*. 2014) or long‐read sequencing (Huddleston *et al*. 2014; Laszlo *et al*. 2014) have proven useful to improve the assembly of complex regions or otherwise finish draft genome sequences.

Many comparisons of our genome annotations (e.g. SNPs, coding sequences, noncoding RNAs) with the current dromedary reference genome were not possible due to the unavailability of these data. Therefore, in congruence with current recommendations for data sharing in ecology and evolution (Whitlock 2011), we have archived all data for this study in various locations (see Data accessibility section below) thus providing extensive resources to the camel‐research community. In addition to the data, we make example bioinformatics code available to promote open, reproducible research and external evaluation as advocated by others (Mesirov 2010; Stodden *et al*. 2010; Peng 2011; Groves & Godlee 2012). The availability of genomic resources for dromedaries will facilitate future evolutionary studies of camels and the application of marker‐assisted breeding selection to improve the yield and performance of camel‐derived products. Because camelids, notably dromedaries, are especially adapted to harsh, arid environments, understanding how the process of natural and artificial selection that has shaped their unique traits has implications in both evolutionary biology and agriculture.

R.R.F. wrote the study and performed bioinformatic analyses. E.M. extracted DNA, performed bioinformatic analyses and revised the manuscript. J.C. provided the extensive computational resources necessary for the completion of the project and revised the manuscript. P.A.B. managed the project, carried out initial raw data analysis and revised the manuscript.

## Data accessibility

Project Information: NCBI BioProject PRJNA269274; NCBI SRA SRP050586.

Sample Information: NCBI BioSample – SAMN03252735; NCBI SRA – SRS779886.

Raw Sequence Reads: NCBI SRA – SRX796513 (500‐bp insert), SRX796571 (5‐kb insert).

Trimmed and Error‐corrected paired‐end reads: NCBI SRA – SRX1013838.

Genome Assembly: GenBank Accession no. GCA_000803125.1.

Read alignments (.bam format): NCBI SRA – SRR1950615.

SNP data, protein and RNA annotations, and the genome alignment are available in Dryad under doi:10.5061/dryad.v28f9.

Example scripts and code: Online Supporting Information, Methods S1 and S2.

## Supporting information


**Fig. S1** The count of unique 31‐mers that are found *n* (multiplicity) times in the trimmed and error‐corrected paired‐end reads (blue line).
**Fig. S2** The count (A) and cumulative proportion (B) of unique 20‐mers that are found *n* (multiplicity) times in the raw, paired‐end sequencing reads (red line) and the trimmed and error‐corrected paired‐end reads (blue line).
**Fig. S3** Histogram of the base quality scores corrected in the forward (green line), reverse (blue line) and unpaired (yellow line) raw reads.
**Fig. S4** Comparison of (A) the number of scaffolds, (B) N50 length, (C) Proportion of 248 core eukaryotic genes (CEGs) annotated, and (D) longest scaffold length for various *k*‐mer sizes used to assemble the genome.
**Fig. S5** Distribution of the species (outer circle) and sequence types (inner circle) for the top blast hit for each of the short scaffolds (<500 bp) omitted from the final assembly.
**Fig. S6** Histogram of the lengths (in base‐pairs) of alignment blocks between our dromedary genome assembly and the reference (Accession no. GCA_000767585.1).
**Fig. S7** Cumulative number of genes ordered by increasing AED (annotation edit distance) scores.
**Fig. S8** The distribution of (A) similarity scores for all the BLAST hits and (B) the species of the top hit for each annotated protein sequence.
**Fig. S9** The number of Gene Ontology (GO) terms mapped to each protein sequence.
**Fig. S10** Histogram of the amino acid identity of single‐copy orthologs between the African dromedary assembly and the *Camelus ferus* (solid line) and *Bos taurus* (dashed line) genome assemblies.
**Fig. S11** The relative abundance of repeat classes in the dromedary genome assembly vs. the Kimura divergence from the consensus, using the combined set of annotated repetitive elements.
**Table S1** Summary statistics of unique 20‐mers in the trimmed sequencing reads.
**Table S2** Summary of the *de novo* assemblies made using both uncorrected and error‐corrected (‐C) reads.
**Table S3** The accession numbers of raw reads for the dromedary reference (Accession no. GCA_000767585.1) downloaded and used for comparison.
**Table S4** Statistics of the *de novo* assembled contigs from the abundant 31‐mers.
**Table S5** Statistics of the repetitive elements identified from *de novo* identification in the sequencing reads and the genome assembly, in addition to the homology‐based search and the combined results.
**Table S6** Summary of the non‐coding RNA annotations in the dromedary genome assembly.
**Methods S1** Example commands used for different analyses in this study.
**Methods S2** Configuration files for the first (A) and second (B) iterations of maker v2.31.6.
