# Supplementary material for: The de novo genome assembly and annotation of a female domestic dromedary of North African origin
Source: Mol Ecol Resour. 2015 Jul 24;16(1):314–24. doi: 10.1111/1755-0998.12443 (PMC4973839; doi:10.1111/1755-0998.12443)
Supplement: Supplementary file 1 — Fig. S1 The count of unique 31‐mers that are found n (multiplicity) times in the trimmed and error‐corrected paired‐end reads (blue line). Fig. S2 The count (A) and cumulative proportion (B) of unique 20‐mers that are found n (multiplicity) times in the raw, paired‐end sequencing reads (red line) and the trimmed and error‐corrected paired‐end reads (blue line). Fig. S3 Histogram of the base quality scores corrected in the forward (green line), reverse (blue line) and unpaired (yellow line) raw reads. Fig. S4 Comparison of (A) the number of scaffolds, (B) N50 length, (C) Proportion of 248 core eukaryotic genes (CEGs) annotated, and (D) longest scaffold length for various k‐mer sizes used to assemble the genome. Fig. S5 Distribution of the species (outer circle) and sequence types (inner circle) for the top blast hit for each of the short scaffolds (<500 bp) omitted from the final assembly. Fig. S6 Histogram of the lengths (in base‐pairs) of alignment blocks between our dromedary genome assembly and the reference (Accession no. GCA_000767585.1). Fig. S7 Cumulative number of genes ordered by increasing AED (annotation edit distance) scores. Fig. S8 The distribution of (A) similarity scores for all the BLAST hits and (B) the species of the top hit for each annotated protein sequence. Fig. S9 The number of Gene Ontology (GO) terms mapped to each protein sequence. Fig. S10 Histogram of the amino acid identity of single‐copy orthologs between the African dromedary assembly and the Camelus ferus (solid line) and Bos taurus (dashed line) genome assemblies. Fig. S11 The relative abundance of repeat classes in the dromedary genome assembly vs. the Kimura divergence from the consensus, using the combined set of annotated repetitive elements. Table S1 Summary statistics of unique 20‐mers in the trimmed sequencing reads. Table S2 Summary of the de novo assemblies made using both uncorrected and error‐corrected (‐C) reads. Table S3 The accession numbers of raw reads for the dromed [file MEN-16-314-s001.docx]

**Supporting Information for Online Publication**

**Supplementary Tables**

**Table S1.** Summary statistics of unique 20-mers in the trimmed sequencing reads. N = number of unique *k*-mers; N­_>3_ = number of 20-mers occurring more than 3 times; N­_<3_ = number of 20-mers occurring less than 3 times; Error rate = N­_<3_ / (20*N); Total count = count of all 20-mers. ­

| N­ | N­_>3_ (True *k*-mers) | N­_<3_ (Error *k*-mers) | Error Rate | Total Count |
| --- | --- | --- | --- | --- |
| 4,022,537,264 | 1,869,966,353 | 2,152,570,911 | 0.027 | 78,706,145,111 |

**Table S2.** Summary of the *de novo* assemblies made using both uncorrected and error-corrected (-C) reads. The “Broken Assembly” is the assembly created using REAPR after splitting scaffolds when an error occurs over a gap, or when an error contains more than one gap.

| Assembly | *k*=48 | *k*=48-C | *k*=64 | *k*=64-C^1^ |
| --- | --- | --- | --- | --- |
| # Scaffolds | 24,645 | 24,058 | 36,126 | 35,752 |
| Mean Length | 81,938.5 | 83,900.2 | 56,881.5 | 57,481.1 |
| Total Length | 2,019,374,771 | 2,018,471,819 | 2,054,899,881 | 2,055,063,633 |
| Longest | 8,065,708 | 5,799,438 | 7,585,309 | 9,719,801 |
| N50 (count) | 1,280,066 (472) | 1,289,612 (469) | 1,481,317 (415) | 1,482,444 (393) |
| N60 (count) | 982,628 (652) | 992,344 (649) | 1,102,948 (577) | 1,108,832 (553) |
| N70 (count) | 740,637 (889) | 740,619 (886) | 827,870 (794) | 842,144 (764) |
| N80 (count) | 512,933 (1,214) | 506,582 (1,214) | 551,192 (1,096) | 558,658 (1,063) |
| N90 (count) | 257,539 (1,755) | 261,637 (1,754) | 254,921 (1,642) | 260,185 (1,592) |
| Number of Gaps | 159,154 | 152,126 | 167,153 | 150,386 |
| Total Gap Length | 65,421,832 | 64,720,402 | 54,478,522 | 53,439,631 |
| Error Free Bases | 89.6% | 89.6% | 91.0% | 91.8% |
| FCD Errors | 48,604 | 48,050 | 44,673 | 37,015 |
| Collapsed Repeats | 8,479 | 8,411 | 10,707 | 10,233 |
| Wrong Read Orientation | 178,184 | 175,162 | 118,250 | 113,677 |
| CEGs^1^ | 99.1% | 99.1% | 98.5% | 98.7% |
| Broken Assembly |  |  |  |  |
| # Scaffolds | 57,049 | 55,758 | 69,307 | 62,302 |
| Mean Length | 35,349.4 | 36,152.5 | 29,603.7 | 32,947.9 |
| Total Length | 2,016,645,998 | 2,015,790,090 | 2,051,743,927 | 2,052,720,440 |
| Longest | 838,048 | 715,267 | 1,293,716 | 1,086,943 |
| N50 (count) | 103,294 (5,956) | 106,329 (5,786) | 150,381 (3,983) | 174,415 (3,471) |
| N60 (count) | 83,435 (8,133) | 85,164 (7,907) | 119,057 (5,514) | 138,871 (4,785) |
| N70 (count) | 64,995 (10,863) | 66,211 (10,585) | 89,475 (7,494) | 105,027 (6,491) |
| N80 (count) | 46,750 (14,500) | 47,705 (14,157) | 61,826 (10,238) | 72,012 (8,841) |
| N90 (count) | 26,601 (20,101) | 27,221 (19,638) | 32,223 (14,702) | 37,287 (12,690) |
| Number of Gaps | 144,064 | 137,838 | 148,697 | 136,068 |
| Total Gap Length | 70,606,453 | 69,951,648 | 58,361,196 | 56,665,254 |

^1^ proportion of 458 core eukaryotic genes (CEGs) identified using CEGMA.

**Table S3.** The accession numbers of raw reads for the dromedary reference (Accession no. GCA_000767585.1) downloaded and used for comparison.

| Insert Size | | |
| --- | --- | --- |
| 170 bp | **500 bp** | **800 bp** |
| SRR1555056 | SRR1555055 | SRR1555057 |
| SRR1555064 | SRR1555078 | SRR1555058 |
| SRR1555066 | SRR1555084 | SRR1555059 |
| SRR1555068 | SRR1555085 | SRR1555061 |
| SRR1555071 | SRR1555091 | SRR1555095 |
| SRR1555073 | SRR1555093 | SRR1555067 |
| SRR1555075 |  | SRR1555069 |
| SRR1555079 |  | SRR1555087 |
| SRR1555083 |  | SRR1555089 |

**Table S4:** Statistics of the *de novo* assembled contigs from the abundant 31-mers.

| Contigs | Size |
| --- | --- |
| ≥31 bp | 83,811 |
| Total Length | 10,037,583 bp |
| ≥1000 bp | 754 |
| Total Length | 1,308,401 bp |
| Longest Contig | 16,414 bp |
| GC % | 38.72 |
| N50 (count) | 124 (15,769) |
| N75 (count) | 74 (42,480) |

**Table S5.** Statistics of the repetitive elements identified from *de novo* identification in the sequencing reads and the genome assembly, in addition to the homology-based search and the combined results.

|  | ***de novo*: reads** | | ***de novo*: assembly** | | **Homology** | | **Combined** | |
| --- | --- | --- | --- | --- | --- | --- | --- | --- |
|  | **Count** | **(%)** | **Count** | **(%)** | **Count** | **(%)** | **Count** | **(%)** |
| **SINEs** | 217,995 | 1.05 | 227,886 | 1.58 | 470,778 | 3.42 | 473,387 | 3.43 |
| **ALUs** | 0 | 0 | 0 | 0 | 7 | 0 | 7 | 0 |
| **MIRs** | 124,243 | 0.62 | 227,886 | 1.58 | 463,899 | 3.38 | 463,927 | 3.38 |
| **LINEs:** | 512,055 | 8.89 | 792,308 | 14.72 | 812,338 | 18.01 | 1,009,426 | 19.28 |
| **LINE1** | 506,055 | 8.87 | 648,810 | 13.42 | 446,105 | 13.30 | 642,633 | 14.57 |
| **LINE2** | 6,000 | 0.01 | 135,151 | 1.23 | 311,570 | 4.10 | 312,130 | 4.10 |
| **L3/CR1** | 0 | 0 | 6,809 | 0.04 | 40,821 | 0.44 | 40,821 | 0.44 |
| **LTR Elements:** | 116,420 | 1.24 | 268,540 | 4.33 | 294,297 | 5.21 | 324,636 | 5.43 |
| **ERVL** | 5,797 | 0.10 | 68,512 | 1.24 | 80,909 | 1.72 | 80,984 | 1.72 |
| **ERVL-MaLRs** | 62,307 | 0.77 | 123,436 | 1.67 | 137,977 | 2.35 | 138,020 | 2.35 |
| **ERV_class I** | 47,897 | 0.36 | 75,572 | 1.40 | 52,135 | 0.85 | 81,938 | 1.07 |
| **ERV_class II** | 419 | 0 | 529 | 0.01 | 153 | 0 | 571 | 0 |
| **DNA Elements:** | 24,454 | 0.20 | 224,646 | 2 | 339,366 | 3.43 | 341,448 | 3.44 |
| **hAT-Charlie** | 0 | 0 | 136,399 | 1.12 | 186,819 | 1.79 | 186,819 | 1.79 |
| **TcMar-Tigger** | 24,439 | 0.20 | 34,089 | 0.50 | 64,838 | 0.80 | 66,902 | 0.81 |
| **Unclassified** | 30,978 | 0.52 | 22,614 | 0.61 | 6,183 | 0.05 | 67,373 | 0.57 |
| **Small RNA** | 0 | 0 | 1,201 | 0.01 | 96,958 | 0.34 | 96,958 | 0.34 |
| **Satellites** | 0 | 0 | 0 | 0 | 264 | 0.00 | 264 | 0.00 |
| **Simple Repeats** | 535,838 | 1.09 | 458,937 | 0.95 | 502,727 | 1.02 | 504,900 | 1.03 |
| **Low Complexity** | 98,997 | 0.24 | 82,953 | 0.20 | 86,933 | 0.21 | 87,448 | 0.21 |
| **Total** |  | **13.23** |  | **24.39** |  | **31.68** |  | **33.72** |

**Table S6.** Summary of the non-coding RNA annotations in the dromedary genome assembly.

| RNA class | Loci | Models |
| --- | --- | --- |
| *cis*-reulatory RNA | 24 | 18 |
| lncRNA | 21 | 21 |
| miRNA | 1,369 | 201 |
| Ribozyme | 1 | 1 |
| rRNA | 45 | 3 |
| snoRNA | 524 | 208 |
| snRNA | 966 | 11 |
| tRNA | 475 | 2 |
| Other | 266 | 178 |
| Total | 3,691 | 643 |

**Figure S1.** The count of unique 31-mers that are found *n* (multiplicity) times in the trimmed and error-corrected paired-end reads (blue line). The black and red dashed lines indicate the threshold for defining abundant 31-mers (*n* > 98) and the linear function fit to the descending region of the curve, respectively.

**Figure S2.** The count (A) and cumulative proportion (B) of unique 20-mers that are found *n* (multiplicity) times in the raw, paired-end sequencing reads (red line) and the trimmed and error-corrected paired-end reads (blue line). The vertical, dashed line indicates the threshold (*n* = 3) separating error *k*-mers (left of vertical line) from true *k*-mers (right of line).

**Figure S3.** Histogram of the base quality scores corrected in the forward (green line), reverse (blue line) and unpaired (yellow line) raw reads.

**Figure S4.** Comparison of (A) the number of scaffolds, (B) N50 length, (C) Proportion of 248 core eukaryotic genes (CEGs) annotated, and (D) longest scaffold length for various *k*-mer sizes used to assemble the genome. The trimmed and error-corrected paired-end reads were used for the assembly and trimmed mate-pair reads for the scaffolding step. Statistics are based upon scaffolds ≥500 bp in length.

**Figure S5.** Distribution of the species (outer circle) and sequence types (inner circle) for the top blast hit for each of the short scaffolds (<500 bp) omitted from the final assembly. 1 = *C. dromedarius* microsatellite sequences, 2 = uncharacterized sequence clones, 3 = other, 4 = genes.

**Figure S6.** Histogram of the lengths (in base-pairs) of alignment blocks between our dromedary genome assembly and the reference (Accession no. GCA_000767585.1).

**Figure S7.** Cumulative number of genes ordered by increasing AED (annotation edit distance) scores. Only annotations with an AED score <0.75 (dashed line) were kept.

**Figure S8.** The distribution of (A) similarity scores for all the BLAST hits and (B) the species of the top hit for each annotated protein sequence. BLAST searches were performed against known metazoan protein sequences from Genbank’s ‘nr’ database. Only the top 20 hits were kept for each gene with a minimum e-value of 10^-3^. In (B), only the 25 most common species are shown.

**Figure S9.** The number of Gene Ontology (GO) terms mapped to each protein sequence.

**Figure S10.** Histogram of the amino acid identity of single-copy orthologs between the African dromedary assembly and the *Camelus ferus* (solid line) and *Bos taurus* (dashed line) genome assemblies.

**Figure S11.** The relative abundance of repeat classes in the dromedary genome assembly versus the Kimura divergence from the consensus, using the combined set of annotated repetitive elements.

**Methods S1.** Example commands used for different analyses in this study. These are provided simply for reference and the reader should consult the software manuals for descriptions of the parameters used.

# Sequence trimming using POPOOLATION v1.2.2 for paired-end and mate-pair reads

trim-fastq.pl \

–input Forward-PE-Reads.fq \

–input Reverse-PE-Reads.fq \

–quality-threshold 20 \

–min-length 50

trim-fastq.pl \

–input Forward-MP-Reads.fq \

–input Reverse-MP-Reads.fq \

–quality-threshold 20 \

–min-length 30

# K-mer counting using DSK v1.6066 (k = 20)

dsk All-PE-Reads.fq 20 -t 1 -o dsk.k22.out -histo

# Error correction using QUAKE v0.3.5

parse_results dsk.k22.out.solid_kmers_binary > dsk_pe.k20.counts

cov_model.py --int dsk_pe.k20.counts

correct -f PE-reads.infile -k 20 -m dsk_pe.k20.counts -c 3 -p 16 –log

# Genome assembly using ABYSS v1.3.6 (e.g. k = 64)

abyss-pe \

v=-v \

np=16 \

k=64 \

n=5 \

s=200 \

name=Drom64K \

lib=’drom’ \

mp=’mp’ \

drom=’Corrected-Forward-PE-Reads.fastq Corrected-Reverse-PE-Reads.fastq’ \

se=’Corrected-SE-Reads.fq’ \

mp=’ Forward-MP-Reads.fastq Reverse-MP-Reads.fastq’

# Assess core eukaryotic gene content using CEGMA v2.4.010312

cegma --mam -o Drom-CEGMA -v -T 16 -g Genome-assembly.fa

Basic quantitative analysis of an assembly using REAPR v1.0.16

smalt index \

-k 13 -s 2 Genome-assembly_index Genome-assembly.fa

smalt sample \

-u 1000 -n 16 -o Genome-assembly_sample \

Genome-assembly_index Corrected-Forward-PE-Reads.fastq Corrected-Reverse-PE-Reads.fastq

smalt map \

-r 0 -x -y 0.5 -n 16 -g Genome-assembly_sample \

-f samsoft \

Genome-assembly_index \

Corrected-Forward-PE-Reads.fastq \

Corrected-Reverse-PE-Reads.fastq | \

awk '$1!~/^#/' | \

samtools view -S -T Genome-assembly.fa -b - > smalt.raw.bam

samtools sort smalt.raw.bam smalt.raw.bam.sort

samtools rmdup smalt.raw.bam.sort.bam smalt.sort.rmdup.bam

echo "@HD VN:1.0 SO:coordinate" | awk '{OFS="\t"; $1=$1; print}' > smalt.header

samtools view -H smalt.sort.rmdup.bam >> smalt.header

samtools reheader smalt.header smalt.sort.rmdup.bam > smalt.final.bam

samtools index smalt.final.bam

reapr pipeline Genome-assembly.fa smalt.final.bam REAPR-OUT

# Align two dromedary genomes using MUGSY v1.2.3

mugsy -p Drom --directory . -d 500 -plot Cdrom-genbank.fa Genome-assembly.fa

# Calculate alignment statistics using MAFFILTER v1.1.0,

maffilter \

input.file=Drom.maf \

input.file.compression=none \

output.log=Drom.maffilter.log \

maf.filter=\

AlnFilter(\

species=(Cdrom_genbank,Drom64K_repmod),\

window.size=10,\

window.step=1,\

max.gap=5,\

missing_as_gap=yes,\

file=data.trash_aln.maf.gz,\

compression=gzip),\

MinBlockSize(min_size=2),\

MinBlockLength(min_length=500),\

Output(\

file=Drom.filtered.maf.gz,\

compression=gzip,\

mask=yes),\

SequenceStatistics(\

statistics=(\

BlockLength(),\

SequenceLength(\

species=Drom64K_repmod),\

SequenceLength(\

species=Cdrom_genbank),\

SiteStatistics(\

species=Drom64K_repmod),\

SiteStatistics(\

species=Cdrom_genbank),\

AlnScore(),\

PairwiseDivergence(\

species1=Cdrom_genbank,\

species2=Drom64K_repmod)),\

ref_species=Cdrom_genbank,\

file=Drom.filtered.statistics.blocks.csv),\

WindowSplit(\

preferred_size=500,\

align=center),\

SequenceStatistics(\

statistics=(\

BlockLength(),\

SequenceLength(\

species=Drom64K_repmod),\

SequenceLength(\

species=Cdrom_genbank),\

SiteStatistics(\

species=Drom64K_repmod),\

SiteStatistics(\

species=Cdrom_genbank),\

AlnScore(),\

PairwiseDivergence(\

species1=Cdrom_genbank,\

species2=Drom64K_repmod)),\

ref_species=Drom64K_repmod,\

file=Drom.filtered.statistics.windows.csv)

# Calculate alignment statistics using MAFFILTER v1.1.0, for features in a GFF file (e.g. CPG Islands)

maffilter \

input.file=Drom.filtered.maf.gz \

input.file.compression=gzip \

output.log=Drom.maffilter.exons.log \

maf.filter=\

MinBlockSize(min_size=2),\

ExtractFeature(\

ref_species=Drom64K_repmod,\

feature.file=exons.gff3,\

feature.file.compression=none,\

feature.format=GFF,\

feature.type=all,\

complete=yes,\

ignore_strand=no),\

SequenceStatistics(\

statistics=(\

BlockLength(),\

SequenceLength(\

species=Drom64K_repmod),\

SequenceLength(\

species=Cdrom_genbank),\

SiteStatistics(\

species=Drom64K_repmod),\

SiteStatistics(\

species=Cdrom_genbank),\

AlnScore(),\

PairwiseDivergence(\

species1=Cdrom_genbank,\

species2=Drom64K_repmod)),\

ref_species=Drom64K_repmod,\

file=Drom.statistics.cpg.csv),\

Output(\

file=Drom.cpg.maf.gz,\

compression=gzip,\

mask=yes)

# Extraction of divergent sites as a .vcf file from the genome alignment using MAFFILTER v1.1.0

maffilter \

input.file=Drom.filtered.maf.gz \

input.file.compression=gzip \

output.log=Drom.maffilter.vcf.log \

maf.filter=\

VcfOutput(\

file=Drom.MAFsnp.vcf.gz,\

compression=gzip,\

reference=Drom64K_repmod,\

genotypes=(\

Drom64K_repmod,Cdrom_genbank))

# Predict genes using GENEMARK-ES

perl gm_es.pl -v Genome-assembly.fa

# Convert CEGMA results into SNAP hidden Markov model

cegma2zff Drom-CEGMA.cegma.gff Genome-assembly.fa

fathom genome.ann genome.dna -categorize 1000

fathom -export 1000 -plus uni.ann uni.dna

forge export.ann export.dna

hmm-assembler.pl drom . > cegmasnap.hmm

# Run first iteration of MAKER v2.31.6 # see Appendix 2A for MAKER configuration file

maker -base MAKER1

# Merge MAKER annotations into a single gff file and build new SNAP model

gff3_merge -d MAKER1.maker.output/MAKER1_master_datastore_index.log -o maker1_All.gff

maker2zff maker1_All.gff

fathom genome.ann genome.dna -categorize 1000

fathom -export 1000 -plus uni.ann uni.dna

forge export.ann export.dna

hmm-assembler.pl maker1 . > snap2.hmm

# Train a model for use with AUGUSTUS v2.5.5

autoAug.pl \

--genome=Genome-assembly.fa \

--species=dromedarius \

--cdna=cDNA.fa \

--trainingset=genome.gff3 \

-v -v –v \

–useexisting

# Run second iteration of MAKER v2.31.6 # see Appendix 2B for MAKER configuration files

maker -base MAKER2

# Assignment of orthologs using ORTHOMCL v2.0 (only comparison with *Bos taurus* is shown)

wget <ftp://ftp.ensembl.org/pub/release-77/fasta/bos_taurus/pep/Bos_taurus.UMD3.1.pep.all.fa.gz>

gunzip Bos_taurus.UMD3.1.pep.all.fa.gz

orthomclAdjustFasta Btau Bos_taurus.UMD3.1.pep.all.fa 1

orthomclAdjustFasta Cdro Drom.longestORFs.faa 1

orthomclFilterFasta . 10 20

makeblastdb -in goodProteins.fasta -dbtype prot -parse_seqids -out goodProteins.fasta

blastp -db goodProteins.fasta -query goodProteins.fasta -outfmt 6 -out blastresults.tsv

mkdir SEQS

mv Cdro.fasta SEQS

mv Btau.fasta SEQS

orthomclBlastParser blastresults.tsv ./SEQS >> similarSequences.txt

mysql -u rfitak –p

DROP DATABASE orthomcl;

create database orthomcl;

exit

orthomclInstallSchema mysql.config mysql.log

orthomclLoadBlast mysql.config similarSequences.txt

orthomclPairs mysql.config pairs.log cleanup=no

orthomclDumpPairsFiles mysql.config

mcl mclInput --abc -I 1.5 -o groups_1.5.txt

orthomclMclToGroups OG1.5_ 1000 < groups_1.5.txt > named_groups_1.5.txt

# Masking the genome with REPEATMASKER v4.0.5

RepeatMasker –pa 16 –gff –xsmall Genome-assembly.fa

# Construct *de novo* repeat library with REPEATMODELER

# and mask the genome with REPEATMASKER

RepeatModeler/BuildDatabase –name Genome-assembly-db Genome-assembly.fa

RepeatModeler -engine ncbi -pa 15 -database Genome-assembly-db

RepeatMasker –pa 16 –gff –xsmall –lib consensi.fa.classified Genome-assembly.fa

# Constructing *de novo* repeat libraries from whole genome reads of dromedary genome

# using REPARK v1.2.2 and subsequent masking of the genome

# using REPEATMODELER and REPEATMASKER

perl RepARK.pl -l All-PE-Reads.fq -p 16 -d -o /RepARK/RepARK_working/

RepeatModeler/BuildDatabase –name Genome-RepArk-db /RepARK/ velvet_repeat_lib/contigs.fa

RepeatModeler -engine ncbi -pa 15 -database Genome-RepArk-db

RepeatMasker –pa 16 –gff –xsmall –lib consensi.fa.classified Genome-assembly.fa

# Generate the repeat landscape using REPEATMASKER v4.0.5

perl \

/RepeatMasker/util/calcDivergenceFromAlign.pl \

-s Genome.fa.masked.cat. \

divsum \

Genome.fa.masked.cat.align.gz

perl \

/RepeatMasker/util/createRepeatLandscape.pl \

-div Genome.fa.masked.cat. \

divsum > Genome_landscape.html

# Annotation of RNA sequences using INFERNAL v1.1 (only 1 Rfam model is shown,

# all families were searched)

GA=$(grep "^GA" RF00001.cm | sed 's/^GA[ ]*//g' | perl -ne '$_=0.85 * $_; print "$_"')

cmsearch \

-Z 5400 \

-T $GA \

RF00001.cm \

--tblout RNA.tbl \

Genome-assembly.fa

# Identification of CpG islands using EMBOSS v6.5.7

perl -ne 'if ($_ =~ m/^>/){print "$_";}else{$_ =~ s/[acgt]/N/g; print "$_";}' \

Drom64K_repmod.fa.masked > Drom64K.hardmasked.fasta

cpgplot \

-sequence Drom64K.hardmasked.fasta \

-outfile Drom64K.cpgplot.out \

-noplot \

-window 100 \

-minlen 200 \

-minoe 0.6 \

-minpc 50 \

-outfeat Drom64K.cpgplot.gff3

# Map the paired-end sequencing reads to the assembled genome using BWA 0.6.2

bwa aln \

-n 0.01 \

-o 1 \

-e 12 \

-d 12 \

-l 32 \

-t 16 \

-I \

Genome-assembly.fa \

Corrected-Forward-PE-Reads.fastq > Fwd.sai

bwa aln \

-n 0.01 \

-o 1 \

-e 12 \

-d 12 \

-l 32 \

-t 16 \

-I \

Genome-assembly.fa \

Corrected-Reverse-PE-Reads.fastq > Rev.sai

bwa sampe \

–r $rg \

Genome-assembly.fa \

Fwd.sai \

Rev.sai \

Corrected-Forward-PE-Reads.fastq \

Corrected-Reverse-PE-Reads.fastq | \

samtools view -u - > Dromedary.bam

# Convert alignments to sorted ‘bam’ format and filter for high-quality, properly paired reads

# using SAMTOOLS v1.1 (‘rmdup’ used SAMTOOLS v0.1.19)

samtools view \

-u \

-q 20 \

-f 0x0002 \

-F 0x0004 \

-F 0x0008 \

Dromedary.bam | \

samtools rmdup - - | \

samtools sort \

-O bam \

-T Drom.sorted - > Drom.sorted.rmdup.mq20.bam

# Call SNPs using SAMTOOLS v1.1

samtools mpileup \

-C50 \

-t DP,DPR,DV,DP4,INFO/DPR,SP \

-uf Genome-assembly.fa \

Drom.sorted.rmdup.mq20.bam | \

bcftools call \

-O v \

-c -M \

-A \

-v - > Drom.samtools.raw.vcf

# Call SNPs using PLATYPUS v0.7.9.1

Platypus.py callVariants \

-o Drom.platypus.raw.vcf \

--refFile=Genome-assembly.fa \

--bamFiles=Drom.sorted.rmdup.mq20.bam \

--nCPU 16

# Reduce MNVs to SNVs using GATK VariantsToAllelicPrimitives

java -Xmx20g -jar GenomeAnalysisTK.jar \

-T VariantsToAllelicPrimitives \

-R Genome-assembly.fa \

--variant Drom.platypus.raw.vcf \

-o Drom.platypus.primitives.vcf

# Keep only SNPs with "PASS" from PLATYPUS variants using VCFTOOLS v.0.1.12

vcftools \

--vcf Drom.platypus.primitives.vcf \

--remove-indels \

--recode \

--recode-INFO-all \

--remove-filtered-all \

--out Drom.platypus.primitives.filtered.vcf

# Find the intersection of raw SAMTOOLS SNPs with the filtered PLATYPUS SNPs

# using BEDTOOLS v.2.17.0

intersectBed \

-wa \

-header \

-a Drom.samtools.rawSNPs.vcf \

-b Drom.platypus.primitives.filtered.vcf > Drom-SAM-PL.overlap.vcf

# Filter and annotate the overlapping SNPs

# using SAMTOOLS/BCFTOOLS v1.1 and VCFTOOLS v.0.1.12

bcftools filter \

-O v \

-g5 \

-G5 \

-i 'QUAL>=20 && DP>=14 && DP<=86' \

-s QUAL-DP < Drom-SAM-PL.overlap.vcf | \

bcftools filter \

-O v \

-i 'INFO/PLATYPUS!="FAIL"' \

-s PLATYPUS \

-m+ - > Drom.overlap.filtered.vcf

vcftools \

--vcf Drom.overlap.filtered.vcf \

--recode \

--recode-INFO-all \

--remove-filtered-all \

--out Drom.final.vcf

# Calculate Ti/Tv ratio using VCFTOOLS v.0.1.12

vcftools \

--vcf Drom.final.vcf \

--remove-indels \

--TsTv-summary \

--out Drom.TiTv

# Calculate SNP density in 1kb windows using VCFTOOLS v.0.1.12

vcftools \

--vcf Drom.final.vcf \

--remove-indels \

--SNPdensity 1000 \

--out Drom

# Calculate SNP density in annotated regions using VCFTOOLS v.0.1.12 (only exons shown)

vcftools \

--vcf Drom.final.vcf \

--remove-indels \

--bed exons.bed \

--recode \

--out Drom.exons

# Demographic history using PSMC and the default parameters,

# with a coverage cutoff minimum of 14x

# and maximum of 86x (1/3 and 2x the mean coverage).

samtools mpileup \

-C50 \

-S \

-D \

-uf Genome-assembly.fa \

Drom.sorted.rmdup.mq20.bam | \

bcftools view \

-c - | \

vcfutils.pl vcf2fq \

-d 14 \

-D 86 | \

gzip > Drom.raw.fq.gz

fq2psmcfa Drom.raw.fq.gz > Drom.raw.psmcfa

splitfa Drom.raw.psmcfa > Drom.raw.split.psmcfa

psmc \

-N25 \

-t15 \

-r5 \

-p "4+25*2+4+6" \

Drom.raw.psmcfa \

-o Drom.raw.psmc

for i in {1..100}; \

do \

psmc \

-N25 \

-t15 \

-r5 \

-b \

-p "4+25*2+4+6" \

-o Drom.raw.$i.psmc \

Drom.raw.split.psmcfa; \

done

cat \

Drom.raw.psmc \

Drom.raw.*.psmc > bootstrapped.raw.psmc

psmc_plot.pl \

-P bottom \

-X2000000 -p \

-g5 \

-x1000 \

Plot.bootstrapped.raw \

bootstrapped.raw.psmc

# Demographic history using PSMC with the repeat-masked genome and the filtered set of SNPs.

bcftools consensus \

-f Genome.fa.masked \

-i Drom.overlap.filtered.vcf > Drom.filtered.fa

fq2psmcfa Drom.filtered.fa > Drom.filtered.psmcfa

splitfa Drom.filtered.psmcfa > Drom.filtered.split.psmcfa

psmc \

-N25 \

-t15 \

-r5 \

-p "4+25*2+4+6" \

Drom.filtered.psmcfa -o Drom.filtered.psmc

for i in {1..100}; \

do \

psmc \

-N25 \

-t15 \

-r5 \

-b \

-p "4+25*2+4+6" \

-o Drom.filtered.$i.psmc \

Drom.filtered.split.psmcfa; \

done

psmc_plot.pl \

-P bottom \

-X2000000 \

-p \

-g5 \

-x1000 \

Plot.bootstrapped.filtered \

bootstrapped.filtered.psmc

**Methods S2.** Configuration files for the first (A) and second (B) iterations of MAKER v2.31.6

A. maker_opts.ctl configuration file for the first iteration of MAKER

#-----Genome (these are always required)

genome=Genome-assembly.fa #genome sequence (fasta file or fasta embeded in GFF3 file)

organism_type=eukaryotic #eukaryotic or prokaryotic. Default is eukaryotic

#-----Re-annotation Using MAKER Derived GFF3

maker_gff= #MAKER derived GFF3 file

est_pass=0 #use ESTs in maker_gff: 1 = yes, 0 = no

altest_pass=0 #use alternate organism ESTs in maker_gff: 1 = yes, 0 = no

protein_pass=0 #use protein alignments in maker_gff: 1 = yes, 0 = no

rm_pass=0 #use repeats in maker_gff: 1 = yes, 0 = no

model_pass=0 #use gene models in maker_gff: 1 = yes, 0 = no

pred_pass=0 #use ab-initio predictions in maker_gff: 1 = yes, 0 = no

other_pass=0 #passthrough anyything else in maker_gff: 1 = yes, 0 = no

#-----EST Evidence (for best results provide a file for at least one)

est=cDNA.fa #set of ESTs or assembled mRNA-seq in fasta format

altest= #EST/cDNA sequence file in fasta format from an alternate organism

est_gff= #aligned ESTs or mRNA-seq from an external GFF3 file

altest_gff= #aligned ESTs from a closly relate species in GFF3 format

#-----Protein Homology Evidence (for best results provide a file for at least one)

protein=homologous-proteins.fa #protein sequence file in fasta format (i.e. from cow, Bactrian camel, and alpaca)

protein_gff= #aligned protein homology evidence from an external GFF3 file

#-----Repeat Masking (leave values blank to skip repeat masking)

model_org=all #select a model organism for RepBase masking in RepeatMasker

rmlib= #provide an organism specific repeat library in fasta format for RepeatMasker

repeat_protein=te_proteins.fasta #provide a fasta file of transposable element proteins for RepeatRunner

rm_gff= #pre-identified repeat elements from an external GFF3 file

prok_rm=0 #forces MAKER to repeatmask prokaryotes (no reason to change this), 1 = yes, 0 = no

softmask=1 #use soft-masking rather than hard-masking in BLAST (i.e. seg and dust filtering)

#-----Gene Prediction

snaphmm=cegmasnap.hmm #SNAP HMM file

gmhmm=es.mod #GeneMark HMM file

augustus_species= #Augustus gene prediction species model

fgenesh_par_file= #FGENESH parameter file

pred_gff= #ab-initio predictions from an external GFF3 file

model_gff= #annotated gene models from an external GFF3 file (annotation pass-through)

est2genome=1 #infer gene predictions directly from ESTs, 1 = yes, 0 = no

protein2genome=1 #infer predictions from protein homology, 1 = yes, 0 = no

trna=1 #find tRNAs with tRNAscan, 1 = yes, 0 = no

snoscan_rrna= #rRNA file to have Snoscan find snoRNAs

unmask=0 #also run ab-initio prediction programs on unmasked sequence, 1 = yes, 0 = no

#-----Other Annotation Feature Types (features MAKER doesn't recognize)

other_gff= #extra features to pass-through to final MAKER generated GFF3 file

#-----External Application Behavior Options

alt_peptide=C #amino acid used to replace non-standard amino acids in BLAST databases

cpus=1 #max number of cpus to use in BLAST and RepeatMasker (not for MPI, leave 1 when using MPI)

#-----MAKER Behavior Options

max_dna_len=100000 #length for dividing up contigs into chunks (increases/decreases memory usage)

min_contig=1 #skip genome contigs below this length (under 10kb are often useless)

pred_flank=200 #flank for extending evidence clusters sent to gene predictors

pred_stats=0 #report AED and QI statistics for all predictions as well as models

AED_threshold=1 #Maximum Annotation Edit Distance allowed (bound by 0 and 1)

min_protein=0 #require at least this many amino acids in predicted proteins

alt_splice=0 #Take extra steps to try and find alternative splicing, 1 = yes, 0 = no

always_complete=0 #extra steps to force start and stop codons, 1 = yes, 0 = no

map_forward=0 #map names and attributes forward from old GFF3 genes, 1 = yes, 0 = no

keep_preds=1 #Concordance threshold to add unsupported gene prediction (bound by 0 and 1)

split_hit=10000 #length for the splitting of hits (expected max intron size for evidence alignments)

single_exon=1 #consider single exon EST evidence when generating annotations, 1 = yes, 0 = no

single_length=250 #min length required for single exon ESTs if 'single_exon is enabled'

correct_est_fusion=0 #limits use of ESTs in annotation to avoid fusion genes

tries=2 #number of times to try a contig if there is a failure for some reason

clean_try=0 #remove all data from previous run before retrying, 1 = yes, 0 = no

clean_up=0 #removes theVoid directory with individual analysis files, 1 = yes, 0 = no

TMP= #specify a directory other than the system default temporary directory for temporary files

B. maker_opts.ctl configuration file for the second iteration of MAKER

#-----Genome (these are always required)

genome=Genome-assembly.fa #genome sequence (fasta file or fasta embeded in GFF3 file)

organism_type=eukaryotic #eukaryotic or prokaryotic. Default is eukaryotic

#-----Re-annotation Using MAKER Derived GFF3

maker_gff= #MAKER derived GFF3 file

est_pass=0 #use ESTs in maker_gff: 1 = yes, 0 = no

altest_pass=0 #use alternate organism ESTs in maker_gff: 1 = yes, 0 = no

protein_pass=0 #use protein alignments in maker_gff: 1 = yes, 0 = no

rm_pass=0 #use repeats in maker_gff: 1 = yes, 0 = no

model_pass=0 #use gene models in maker_gff: 1 = yes, 0 = no

pred_pass=0 #use ab-initio predictions in maker_gff: 1 = yes, 0 = no

other_pass=0 #passthrough anyything else in maker_gff: 1 = yes, 0 = no

#-----EST Evidence (for best results provide a file for at least one)

est=cDNA.fa #set of ESTs or assembled mRNA-seq in fasta format

altest= #EST/cDNA sequence file in fasta format from an alternate organism

est_gff= #aligned ESTs or mRNA-seq from an external GFF3 file

altest_gff= #aligned ESTs from a closly relate species in GFF3 format

#-----Protein Homology Evidence (for best results provide a file for at least one)

protein=homologous-proteins.fa #protein sequence file in fasta format (i.e. from cow, Bactrian camel, alpaca)

protein_gff= #aligned protein homology evidence from an external GFF3 file

#-----Repeat Masking (leave values blank to skip repeat masking)

model_org=all #select a model organism for RepBase masking in RepeatMasker

rmlib= #provide an organism specific repeat library in fasta format for RepeatMasker

repeat_protein=te_proteins.fasta #provide a fasta file of transposable element proteins for RepeatRunner

rm_gff= #pre-identified repeat elements from an external GFF3 file

prok_rm=0 #forces MAKER to repeatmask prokaryotes (no reason to change this), 1 = yes, 0 = no

softmask=1 #use soft-masking rather than hard-masking in BLAST (i.e. seg and dust filtering)

#-----Gene Prediction

snaphmm=snap2.hmm #SNAP HMM file

gmhmm=es.mod #GeneMark HMM file

augustus_species=dromedarius #Augustus gene prediction species model

fgenesh_par_file= #FGENESH parameter file

pred_gff= #ab-initio predictions from an external GFF3 file

model_gff= #annotated gene models from an external GFF3 file (annotation pass-through)

est2genome=0 #infer gene predictions directly from ESTs, 1 = yes, 0 = no

protein2genome=0 #infer predictions from protein homology, 1 = yes, 0 = no

trna=1 #find tRNAs with tRNAscan, 1 = yes, 0 = no

snoscan_rrna= #rRNA file to have Snoscan find snoRNAs

unmask=0 #also run ab-initio prediction programs on unmasked sequence, 1 = yes, 0 = no

#-----Other Annotation Feature Types (features MAKER doesn't recognize)

other_gff= #extra features to pass-through to final MAKER generated GFF3 file

#-----External Application Behavior Options

alt_peptide=C #amino acid used to replace non-standard amino acids in BLAST databases

cpus=1 #max number of cpus to use in BLAST and RepeatMasker (not for MPI, leave 1 when using MPI)

#-----MAKER Behavior Options

max_dna_len=100000 #length for dividing up contigs into chunks (increases/decreases memory usage)

min_contig=1 #skip genome contigs below this length (under 10kb are often useless)

pred_flank=200 #flank for extending evidence clusters sent to gene predictors

pred_stats=1 #report AED and QI statistics for all predictions as well as models

AED_threshold=1 #Maximum Annotation Edit Distance allowed (bound by 0 and 1)

min_protein=30 #require at least this many amino acids in predicted proteins

alt_splice=1 #Take extra steps to try and find alternative splicing, 1 = yes, 0 = no

always_complete=0 #extra steps to force start and stop codons, 1 = yes, 0 = no

map_forward=0 #map names and attributes forward from old GFF3 genes, 1 = yes, 0 = no

keep_preds=1 #Concordance threshold to add unsupported gene prediction (bound by 0 and 1)

split_hit=10000 #length for the splitting of hits (expected max intron size for evidence alignments)

single_exon=1 #consider single exon EST evidence when generating annotations, 1 = yes, 0 = no

single_length=250 #min length required for single exon ESTs if 'single_exon is enabled'

correct_est_fusion=0 #limits use of ESTs in annotation to avoid fusion genes

tries=2 #number of times to try a contig if there is a failure for some reason

clean_try=0 #remove all data from previous run before retrying, 1 = yes, 0 = no

clean_up=0 #removes theVoid directory with individual analysis files, 1 = yes, 0 = no

TMP= #specify a directory other than the system default temporary directory for temporary files
